# Wearable devices can predict the outcome of standardized 6-minute walk tests in heart disease

**DOI:** 10.1038/s41746-020-0299-2

**Published:** 2020-07-09

**Authors:** Charlotte Schubert, Gareth Archer, Jo M. Zelis, Sarah Nordmeyer, Kilian Runte, Anja Hennemuth, Felix Berger, Volkmar Falk, Pim A. L. Tonino, Rod Hose, Herman ter Horst, Titus Kuehne, Marcus Kelm

**Affiliations:** 1 Institute for Imaging Science and Computational Modelling in Cardiovascular Medicine, Charité-Universitätsmedizin Berlin, 13353 Berlin, Germany; 2 Department of Congenital Heart Disease, German Heart Center Berlin (Deutsches Herzzentrum Berlin, DHZB), 13353 Berlin, Germany; 3 Cardiothoracic Centre, Sheffield Teaching Hospital NHS Foundation Trust, Sheffield, S5 7AT UK; 4 Department of Cardiology, Catharina Hospital Eindhoven, 5602 ZA Eindhoven, The Netherlands; 5 DZHK (German Centre for Cardiovascular Research), Partner Site Berlin, 10785 Berlin, Germany; 6 Department of Cardiothoracic and Vascular Surgery, German Heart Center Berlin (Deutsches Herzzentrum Berlin, DHZB), 13353 Berlin, Germany; 7 Department of Cardiovascular Surgery, Charité-Universitätsmedizin Berlin, 13353 Berlin, Germany; 8 Department of Health Science and Technology, Swiss Federal Institute of Technology, 8092 Zurich, Switzerland; 9 Department of Chronic Disease Management, Philips Electronics Nederland B.V., 5656 AE Eindhoven, The Netherlands; 10 Berlin Institute of Health (BIH), 10178 Berlin, Germany

**Keywords:** Valvular disease, Diagnosis

## Abstract

Wrist-worn devices with heart rate monitoring have become increasingly popular. Although current guidelines advise to consider clinical symptoms and exercise tolerance during decision-making in heart disease, it remains unknown to which extent wearables can help to determine such functional capacity measures. In clinical settings, the 6-minute walk test has become a standardized diagnostic and prognostic marker. We aimed to explore, whether 6-minute walk distances can be predicted by wrist-worn devices in patients with different stages of mitral and aortic valve disease. A total of *n* = 107 sensor datasets with 1,019,748 min of recordings were analysed. Based on heart rate recordings and literature information, activity levels were determined and compared to results from a 6-minute walk test. The percentage of time spent in moderate activity was a predictor for the achievement of gender, age and body mass index-specific 6-minute walk distances (*p* < 0.001; *R*^2^ = 0.48). The uncertainty of these predictions is demonstrated.

## Introduction

The 6-minute walk test is a widely used measure of exercise tolerance and a predictor of patient-centred outcomes. In patients with cardiovascular disease, including valve disease, current guidelines advise considering exercise capacity for diagnostics and treatment planning^1,2^. Wrist-worn devices are constantly improving and have become available to large parts of the population. Today’s sensors typically include mechanical and optical methods to measure activity and heart rate that provide information on individual exercise intensities and gross energy expenditure.

Previous studies have identified wrist-worn devices, accelerometers and pedometers as effective tools to increase patients' daily activity^3,4^ and have explored the associations between physical activity, cardiovascular events and risk factors^5–8^. Whereas many devices are designed to record activity, it has not been studied if wrist-worn devices can predict 6-minute walk tests to accurately assess exercise capacity and enable comparisons between patients.

The 6-minute walk test has been clinically validated and has been used to determine the effects of therapeutic interventions^9,10^ and prognosis^11,12^. Although standardized medical exercise tests such as 6-minute walk tests are easy to perform, they still require visiting healthcare services. Wrist-worn devices could offer the advantage of broad availability and may allow performing measurements at home and during everyday activity. Additionally, wearable devices can provide continuous monitoring which enables trends to be identified, making it easier to distinguish the deteriorating patient from the patient that is doing well.

We, therefore, aimed to analyse if 6-minute walk test results can be predicted by heart rate-based activity profiles obtained from wrist-worn devices in combination with literature data in patients with valvular heart disease.

## Results

### Baseline characteristics and differences between centres

**Fig. 1:**
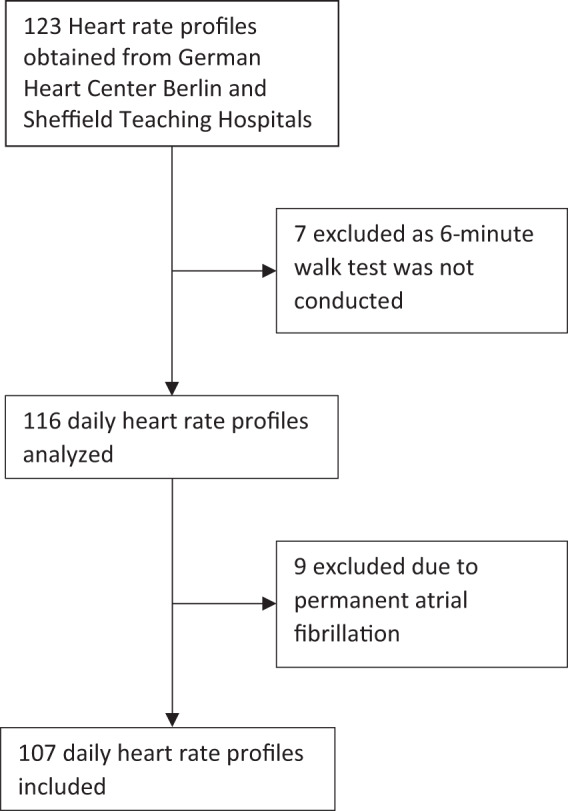
STROBE (Strengthening the Reporting of Observational Studies in Epidemiology Initiative) flow diagram. Overview illustrating the selection of datasets and reasons for exclusion.

In total, *N* = 123 datasets from 91 patients with mitral or aortic valve disease were acquired between March 2017 and October 2018. Of those, *n* = 9 datasets were excluded due to tachycardic atrial fibrillation, and *n* = 7 due to unavailability of 6-minute walk test data, resulting in a total of *n* = 107 included datasets and 1,019,748 min of recordings from 84 patients at both sites (Fig. 1). Accordingly, the average recording time of one dataset was 159 h. A total of *n* = 23 patients contributed a second dataset at the time of a 6-month clinical follow-up after undergoing a valve replacement procedure. As patient characteristics had changed, these patients are represented by two datasets. Baseline characteristics of included datasets are shown in Table 1. Disease severity according to classification standards was mild in 70 (65.4%), moderate in 18 (16.8%) and severe in 19 (17.8%) patients. From all heart rate-based activity levels, time was mostly spent in light activity (51.1%, interquartile range (IQR) 44.8–55.9%). Median 6-minute walk distances were 517 m (IQR 409–581 m). The median percentages of achieved target 6-minute walk distances were 97% (IQR 83.69–109.78%). Targets are patient-specific based on patient age, gender and body mass index (BMI). Parameters of activity are shown in Table 2.

**Table 1:** Baseline characteristics.

Parameter	*N* = 107
Age (years)	66 (56–75)
Male sex (*n*)	65 (60.7%)
Body mass index (kg/m²)	26.82 (24.38–29.53)
Disease severity (*n*)	
None/mild	70 (65.4%)
Moderate	18 (16.8%)
Severe	19 (17.8%)
New York Heart Association class (*n*)	
I	46 (43%)
II	37 (34.6%)
III	18 (16.8%)
IV	3 (2.8%)
Hypertension (*n*)	67 (62.6%)
Diabetes mellitus (*n*)	11 (10.3%)
Smoker (*n*)	15 (14%)
Previous smoker (*n*)	44 (41.1%)
Intermittent atrial fibrillation (*n*)	11 (10.3%)
Permanent atrial fibrillation (*n*)	12 (11.2%)
Beta blockers (*n*)	58 (54.2%)
Diuretics (*n*)	47 (44%)
ARB or ACE-I (*n*)	59 (55.2%)
Calcium antagonists (*n*)	13 (12.1%)
WHOQOL-D1(Physical)	53 (44–56)
WHOQOL-D2 (Psychological)	63 (56–69)
WHOQOL-D3 (Social relationships)	69 (56–81)
WHOQOL-D4 (Environment)	75 (69–88)
Aortic valve disease (*n*)	38 (35.5%)
Mitral valve disease (*n*)	69 (64.5%)
	*N* = 38
Surgical aortic valve replacement (*n*) Transcatheter aortic valve replacement (*n*)	21 (55.3%) 5 (13.2%)
	*N* = 69
Mitral valve repair (*n*)	41 (59.4%)
Mitral valve replacement (*n*)	2 (2.9%)

Data are expressed as median (interquartile range) or absolute numbers (percentage).

*ARB* angiotensin II receptor blockers, *ACE-I* angiotensin-converting enzyme inhibitors, *WHOQOL* World Health Organization Quality of Life BREF score.

**Table 2:** Parameters of activity.

Parameter	*N* = 107
Steps per day	26,258 (20,674.7–34,160.5)
Activity count per day	2,933,540 (2,329,200–3,573,280)
Light activity (%)	51.1 (44.8–55.9)
Moderate activity (%)	0.8 (0.3–1.5)
High activity (%)	0 (0–0.1)
Sleep/rest (%)	47.4 (42.9–52.9)
Activity heart rate index	0.05 (0.03–0.06)
Six-minute walk distance (m)	517 (409–581)
Achieved percentage of target 6-minute walk distance (%)	96.71 (83.69–109.78)

Data are expressed as median (interquartile range) or absolute numbers (percentage).

Of all included datasets, 55 originated from German Heart Centre Berlin and 52 from Sheffield Teaching Hospitals. Patients from both study centres did not differ in daily time spent in different levels of activity and disease severity. Patients from the German Heart Centre Berlin were younger (62.15 vs. 68.38 years, *p* = 0.012), had lower BMIs (26.39 vs. 28.19 kg/m^2^, *p* = 0.018), higher number of steps per day (29,883.98 vs. 24,931.41 steps, *p* = 0.01), higher 6-minute walk distances (554.27 vs. 420.13 m, *p* < 0.001) and achieved higher percentages of their target 6-minute walk distances in meters (103.68% vs. 87.16%, *p* < 0.001).

### Correlation between heart rate- and motion sensor-based activity counts

**Fig. 2:**
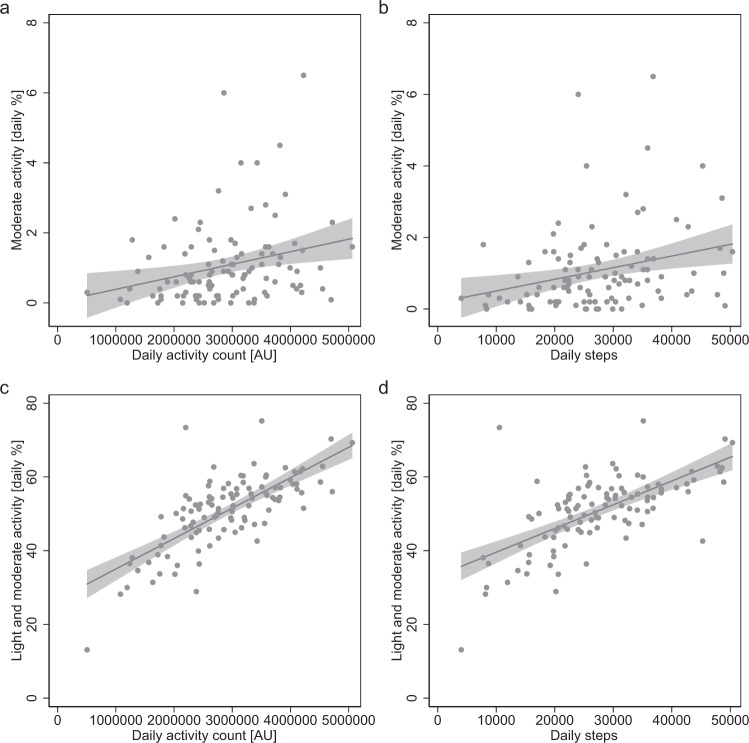
Relationship between steps and activity counts, recorded by the device at different heart rate-based activity levels. **a** Daily activity counts measured by the wrist-worn device plotted against daily time spent in moderate activity (*R*^2^ = 0.087, *p* = 0.002). **b** Daily steps plotted against daily time spent in moderate activity (*R*^2^ = 0.08, *p* = 0.003). **c** Daily activity counts plotted against time spent in combined light and moderate activity (*R*^2^ = 0.539, *p* < 0.001). **d** Daily steps plotted against time spent in combined light and moderate activity (*R*^2^ = 0.422, *p* < 0.001). AU arbitrary units.

Initially, associations between pairs of activity measures were evaluated. Correlations were found between heart rate-based combined daily time spent in light/moderate activity and steps (*R*^2^ = 0.422, *p* < 0.001, Fig. 2d) as well as activity counts (*R*^2^ = 0.539, *p* < 0.001, Fig. 2c), both obtained from motion sensor data of the device. There were also correlations between heart rate-based daily time spent in moderate activity alone and step counts (*R*^2^ = 0.08, *p* = 0.003, Fig. 2b) as well as activity counts (*R*^2^ = 0.087, *p* = 0.002, Fig. 2a).

**Fig. 3:**
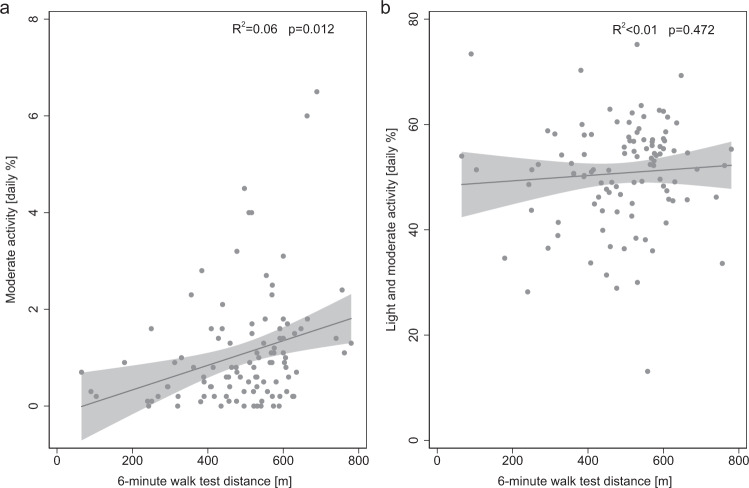
Relationship between 6-minute walk distances and the percent time spent at different heart rate-based activity levels (without consideration of anthropometric and demographic parameters). **a** Six-minute walk distances in meters plotted against daily time spent in moderate activity (*R*^2^ = 0.059, *p* = 0.012). **b** Six-minute walk distances in meters plotted against time spent in combined light and moderate activity (*R*^2^ = 0.005, *p* = 0.472). m meters.

The percentage of time spent in moderate activity correlated with the absolute 6-minute walk distances (*R*^2^ = 0.059, *p* = 0.012, Fig. 3a). No correlations were found between combined time spent in light/moderate activity and the 6-minute walk distances (*R*^2^ = 0.005, *p* = 0.472, Fig. 3b).

For the correlations shown in Fig. 3, other patient-specific data (anthropometrics, demographics) were not considered, and thus they only assess the correlation between pairs of variables.

### Prediction of 6-minute walk test outcomes

**Fig. 4:**
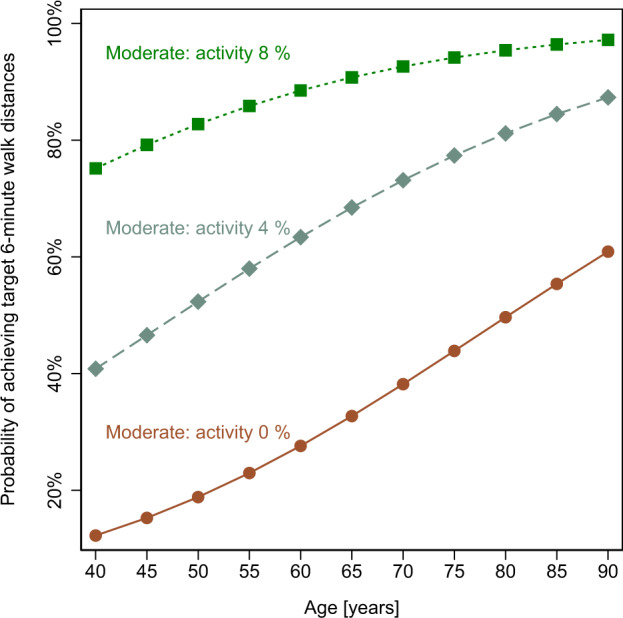
Probabilities of achieving target 6-minute walk distances dependent on age. Curves represent different percentages (0, 4 and 8%) of daily time spent in moderate activity.

In a logistic regression model, the combination of the time spent in moderate activity, age and the type of disease were predictors for the achievement of patient-specific target 6-minute walk distances (overall model’s *p* < 0.001 (pseudo-) *R*^2^ = 0.16). The odds ratios were 1.54 (95% CI 1.04–2.3, *p* = 0.037) for percent time spent in moderate activity, 1.05 (95% CI 1.01–1.1, *p* = 0.007) for each year in age, 0.1 (95% CI 0.02–0.56, *p* = 0.009) for presence of aortic stenosis and 0.27 (95% 0.09–0.82, *p* = 0.021) for presence of mitral regurgitation. The area under the ROC curve was 75.9%, with 71% of all cases correctly classified regarding achievement or failure to achieve target 6-minute walk distances (reference distances of healthy individuals), resulting in a sensitivity of 65% and a specificity of 77% for the combined model. The use of beta blockers (95% CI 0.38–2.6, *p* = 0.985) as well as NYHA classes (NYHA II 95% CI 0.67–6.38, *p* = 0.204, NYHA III 95% CI 0.67–6.38, *p* = 0.341, NYHA IV 95% CI 0.05–24.26, *p* = 0.972) did not show a relevant influence within the model. Moreover, running the model without including information on the percentage of time spent in moderate activity did not result in a valid prediction. The probability of achieving target 6-minute walk distances for different percentages of time spent in moderate activity is shown in Fig. 4.

In a robust regression model the time spent in moderate activity in combination with gender, age and BMI as covariates were able to predict 6-minute walk distances (*p* < 0.001, *R*^2^ = 0.48). Each additional percentage of moderate activity led to an increase in 6-minute walk distance of 10.86 m (95% CI 1.38–20.44, *p* = 0.027), every additional year of life resulted in a decrease in 6-minute walk distances (−4.92 m, 95% CI −6.71 to −3.12, *p* < 0.001) and each kg/m^2^ of BMI in a decrease of −7.42 m (95% CI −12.45 to −2.39, *p* = 0.004). On average, women achieved 77.2 m (95% CI −121.96 to −32.43, *p* = 0.001) less than men. Beta blocker therapy was included in the model as a baseline covariate and was without relevant impact on patient-specific 6-minute walk distances (95% CI −32–54 to 42.68, *p* = 0.79). Compared to the minimally detectable changes (MDC) of the 6-minute walk test at 95% confidence intervals in frail older adults of 28.1 m^13^, a systematic review reporting the minimal clinically important difference of the test to range from 14 to 30.5 m^14^, the standard deviations of our model-based predictions were between 12.53 and 49.98 m in men and between 18.72 and 57.83 m in women. According to the time spent in moderate activity, gender, age and BMI, specific predicted 6-minute walk distances in meters (including their uncertainty) are provided in Tables 3 and 4.

**Table 3:** Predicted 6-minute walk distances and standard deviations in meters for men.

Age												
Moderate daily activity	BMI (kg/m^2^)	40 years	45 years	50 years	55 years	60 years	65 years	70 years	75 years	80 years	85 years	90 years
0%	20	684.65 ± 30.36	660.07 ± 27.55	635.48 ± 25.34	610.89 ± 23.58	586.31 ± 22.72	561.72 ± 23.74	537.14 ± 23.64	512.55 ± 25.33	488 ± 27.66	463.38 ± 30.49	438.8 ± 33.68
	30	610.42 ± 21.56	585.83 ± 18.31	561.24 ± 15.7	536.66 ± 14.09	512.07 ± 13.84	487.79 ± 15.02	463 ± 17.33	438.31 ± 20.4	413.73 ± 23.93	389.14 ± 27.75	364.56 ± 31.76
	40	536.18 ± 35.98	511.59 ± 34.61	487.01 ± 34.61	462.42 ± 33.58	437.83 ± 33.97	413.25 ± 34.94	388.66 ± 35.45	388.66 ± 36.45	339.49 ± 40.83	314.91 ± 43.56	290.32 ± 46.57
2%	20	706.37 ± 32.18	681.79 ± 29.31	657.21 ± 26.91	632.62 ± 25.09	608.04 ± 24	583.45 ± 23.73	558.86 ± 23.73	534.28 ± 25.7	509.69 ± 27.75	485.11 ± 30.35	460.52 ± 33.35
	30	632.14 ± 22.01	607.55 ± 18.47	582.97 ± 15.56	558.8 ± 13.32	533.8 ± 12.53	509.21 ± 13.31	484.63 ± 15.44	460.04 ± 17.98	435.46 ± 22	410.87 ± 25.83	386.28 ± 29.86
	40	557.91 ± 34.93	533.32 ± 33.31	508.73 ± 32.25	484.15 ± 31.81	459.56 ± 32	434.98 ± 32.83	410.39 ± 34.32	385.8 ± 35.19	361.22 ± 38.51	336.63 ± 41.23	312.05 ± 44.24
4%	20	728.17 ± 36.56	703.52 ± 33.8	678.93 ± 31.59	654.35 ± 29.82	629.76 ± 28.67	605.18 ± 28.21	580.59 ± 28.47	556.01 ± 29.42	531.42 ± 31.02	506.83 ± 33.02	482.25 ± 35.74
	30	653.87 ± 26.28	629.28 ± 23.11	604.7 ± 20.44	580.11 ± 17.76	555.53 ± 17.57	530.94 ± 16.75	506.35 ± 19.05	481.76 ± 21.24	457.18 ± 24.1	432.6 ± 27.4	408.01 ± 31.01
	40	579.63 ± 36.49	555.05 ± 34.75	530.56 ± 33.54	505.88 ± 32.91	481.29 ± 32.89	456.7 ± 33.49	432.12 ± 34.67	407.53 ± 36.78	382.95 ± 38.54	358.36 ± 41.1	333.77 ± 44
6%	20	749.83 ± 42.71	725.25 ± 40.35	700.66 ± 38.18	676.08 ± 36.55	651.49 ± 35.43	626.9 ± 34.86	602.32 ± 34.87	577.73 ± 34.87	553.15 ± 36.61	528.56 ± 36.61	503.97 ± 40.35
	30	675.59 ± 32.92	651.01 ± 30.22	626.43 ± 27.99	601.84 ± 26.36	577.25 ± 25.44	552.67 ± 25.3	528.08 ± 25.96	503.5 ± 27.36	478.91 ± 27.36	454.32 ± 31.95	429.74 ± 34.9
	40	601.36 ± 40.37	576.77 ± 38.63	552.19 ± 37.36	527.6 ± 36.6	503.02 ± 36.41	478.43 ± 36.76	453.84 ± 37.66	429.26 ± 39.17	404.67 ± 40.93	380.09 ± 43.18	355.5 ± 45.77
8%	20	771.56 ± 49.98	746.98 ± 47.98	722.39 ± 45.88	697.81 ± 43.3	673.22 ± 43.3	648.63 ± 42.67	624.05 ± 42.52	599.5 ± 42.85	574.87 ± 43.65	550.29 ± 44.89	525.7 ± 46.54
	30	697.32 ± 40.78	672.74 ± 38.46	648.15 ± 35.12	623.57 ± 35.12	598.98 ± 33.93	574.39 ± 34.23	549.81 ± 34.23	525.22 ± 35.11	500.64 ± 36.54	476.05 ± 38.44	451.46 ± 40.76
	40	623.31 ± 45.99	598.5 ± 44.32	573.92 ± 43.05	549.33 ± 42.24	524.74 ± 41.9	500.16 ± 42.05	475.57 ± 42.68	450.99 ± 43.77	426.4 ± 45.29	401.81 ± 47.19	377.23 ± 49.43

*BMI* body mass index, *kg* kilogram, *m* meters.

**Table 4:** Predicted 6-minute walk distances and standard deviations in meters for women.

Age												
Moderate daily activity	BMI (kg/m^2^)	40 years	45 years	50 years	55 years	60 years	65 years	70 years	75 years	80 years	85 years	90 years
0%	20	607.46 ± 38.84	582.87 ± 35.75	558.28 ± 35.75	533.7 ± 30.64	509.11 ± 28.81	484.53 ± 27.63	459.94 ± 27.14	435.35 ± 27.4	410.77 ± 28.38	386.18 ± 30.03	361.6 ± 32.23
	30	533.22 ± 30.52	508.63 ± 27.19	484.73 ± 24.01	459.46 ± 21.55	434.88 ± 19.62	410.29 ± 18.72	385.7 ± 18.89	361.12 ± 20.4	336.53 ± 22.19	311.95 ± 24.93	287.36 ± 28.13
	40	458.98 ± 40.5	434.4 ± 38.41	409.81 ± 36.76	385.22 ± 35.61	360.64 ± 35.01	336.05 ± 34.99	311.47 ± 35.55	286.88 ± 36.67	262.29 ± 38.28	237.71 ± 40.34	213.12 ± 42.79
2%	20	629.13 ± 41.09	604.6 ± 37.99	580.01 ± 35.21	555.43 ± 32.82	530.84 ± 30.9	506.25 ± 29.56	481.67 ± 28.87	457.08 ± 28.86	432.5 ± 29.59	407.91 ± 30.95	383.32 ± 32.88
	30	554.95 ± 31.87	530.36 ± 28.36	505.77 ± 25.17	481.19 ± 22.44	456.6 ± 20.36	432.02 ± 19.15	407.43 ± 18.96	382.84 ± 19.82	358.26 ± 21.62	333.67 ± 24.14	309.09 ± 27.19
	40	480.71 ± 40.37	456.12 ± 38.1	431.54 ± 36.25	406.95 ± 34.89	382.37 ± 33.08	357.78 ± 33.85	333.19 ± 34.23	308.61 ± 35.19	284.02 ± 36.7	259.44 ± 38.66	234.85 ± 41.04
4%	20	650.91 ± 45.32	626.32 ± 42.32	601.74 ± 39.72	577.15 ± 37.41	552.57 ± 35.57	527.98 ± 34.21	503.4 ± 33.42	478.81 ± 33.22	454.22 ± 33.64	429.64 ± 34.65	405.05 ± 35.19
	30	576.67 ± 35.87	552.09 ± 32.58	527.5 ± 29.61	502.92 ± 27.18	478.33 ± 25.12	453.74 ± 23.89	429.19 ± 23.42	404.57 ± 23.84	379.97 ± 24.1	355.4 ± 27.04	330.81 ± 29.56
	40	502.44 ± 42.5	477.85 ± 40.18	453.26 ± 38.25	428.68 ± 36.78	404.09 ± 35.82	379.51 ± 35.41	354.92 ± 35.59	330.34 ± 36.32	305.75 ± 37.6	281.16 ± 39.35	256.58 ± 41.1
6%	20	672.64 ± 51.04	648.05 ± 48.3	623.47 ± 45.85	598.88 ± 43.73	574.29 ± 41.99	549.71 ± 40.67	525.12 ± 39.84	500.54 ± 39.5	475.95 ± 39.61	451.36 ± 40.37	426.79 ± 41.54
	30	598.4 ± 41.76	573.81 ± 38.8	549.23 ± 36.15	524.05 ± 33.91	500.06 ± 32.15	475.47 ± 30.96	450.89 ± 30.5	426.3 ± 30.5	401.71 ± 31.27	377.13 ± 31.95	352.54 ± 34.65
	40	524.16 ± 46.57	499.58 ± 44.31	474.99 ± 42.41	450.41 ± 40.92	425.82 ± 39.89	401.32 ± 39.36	376.65 ± 39.34	35306 ± 39.34	327.48 ± 40.38	302.89 ± 42.29	278.3 ± 44.16
8%	20	694.36 ± 57.83	669.78 ± 55.3	645.19 ± 53.04	620.61 ± 51.09	596.94 ± 49.47	571.44 ± 48.22	546.85 ± 47.37	522.26 ± 46.94	497.68 ± 46.95	473.09 ± 47.39	448.51 ± 48.25
	30	620.13 ± 48.87	595.54 ± 46.21	570.96 ± 43.86	546.37 ± 41.87	521.78 ± 40.29	497.2 ± 39.17	472.61 ± 38.55	448.03 ± 38.46	423.44 ± 38.89	398.85 ± 39.84	374.27 ± 40.76
	40	545.89 ± 52.14	521.3 ± 50	496.72 ± 48.17	472.13 ± 46.72	447.55 ± 45.67	422.96 ± 45.16	398.38 ± 44.89	373.79 ± 45.18	349.2 ± 45.91	324.62 ± 47.06	300–03 ± 48.62

*BMI* body mass index, *kg* kilogram, *m* meters.

## Discussion

**Fig. 5:**
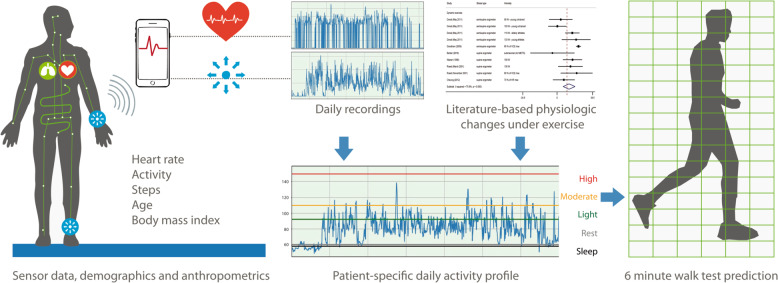
Graphical summary. Step-by-step presentation of the concept for predicting 6-minute walk distances based on daily recordings from wrist-worn devices in combination with demographic and anthropometric data.

In this study, we used heart rate monitoring from wearables in combination with literature-based reference data to determine the daily amount of time spent in different levels of activity. The time spent in moderate activity was able to predict outcomes of a 6-minute walk test in patients with valvular heart disease. In combination with information on a patient’s gender, age, BMI and disease type, absolute 6-minute walk test distances as well as the probability of achieving target 6-minute walk distances can be predicted (Fig. 5). Furthermore, the uncertainty of these model-based predictions is demonstrated and overlapped with the minimal detectable changes and the minimal clinically important differences of the 6-minute walk test.

Exercise testing in cardiology can help to distinguish symptomatic patients, provide prognostic information before therapeutic interventions and thus can play an integral role in decision-making processes^15^. The 6-minute walk test is an inexpensive and feasible method to be performed in the clinical and ambulatory setting. Nevertheless, it is limited to submaximal exercise levels and does not provide information on causes of limiting factors, which has remained a more exclusive domain of ergometric tests^16^. However, especially with bicycle ergometers, maximal exercise levels may not be achieved due to general exhaustion or fatigue of the quadriceps muscle^17^. Both the 6-minute walk test and ergometric exercise tests typically require special equipment and trained personnel and are vastly limited in children and patients with frailty. Both methods, furthermore, strongly depend on the patient's motivation.

In aortic stenosis and mitral regurgitation, a decrease of exercise capacity can indicate the onset of symptoms as well as a worsening of the haemodynamic status and it is therefore commonly regarded as an indication for intervention^1,2^. Its early recognition can be an important determinant for the outcome, as arrhythmia, sudden cardiac death and heart failure can occur when symptomatic patients are left untreated^18–21^. Hence, additional ways for an uncomplicated evaluation of exercise capacity are of potential clinical value.

Activity in this study was identified using daily heart rate profiles obtained by a wrist-worn device. Previous studies assessing the accuracy of such devices have found an overall high accuracy for measuring heart rate^22,23^ as well as steps^24–26^, whereas different intensity levels^24^ and energy expenditure^25,26^ could only be determined imprecisely. The combined time spent in light/moderate activity correlates with daily steps (Fig. 2d). However, the results of the present study indicate that solely determining overall physical activity is not sufficient to predict exercise capacity. Only with quantification of the specific time spent in moderate activity, 6-minute walk test outcomes were effectively predicted. In contrast, the combined time spent in light/moderate activity is dominated by light activity. These measures did not show a correlation to the 6-minute walk distances and even when combined with demographic and anthropometric data it was unable to determine these outcomes. In our study cohort, the probability of achieving target 6-minute walk test goals of a reference population increased with age (Fig. 4). This effect may at least in parts be attributed to shorter overall target distances in the elderly in combination with a tendency for less activity. At the same time, older patients of our study cohort performed better than younger patients when compared to clinically used reference populations of the same age^16^. Nevertheless, patients with a higher percentage of moderate activity performed better within their age group. This also underlines that anthropometric and demographic data alone cannot accurately predict BMI, age and gender-specific 6-minute walk test outcomes in a cohort of patients with heart disease, as individual conditions are not considered. Therefore, the robust regression model combines moderate activity with demographic and anthropometric data to predict individual 6-minute walk distances. High activity occurred only sporadically within the observed patient population and can also include errors due to phases of tachycardia, such as in atrial fibrillation.

Besides identifying arrhythmic events^27^, a heart rate-based approach for evaluating exercise capacity may have the advantage of enabling continuous surveillance during everyday life, e.g., for telemonitoring. A large amount of data gained from telemonitoring could, in turn, be used to further improve the method and reduce its statistical uncertainty. The advantages of continuous monitoring of exercise capacity may be particularly well illustrated by the example of asymptomatic aortic stenosis, where the timing of intervention is still controversial^21,28–30^. While current guidelines strongly advise for surgical intervention as soon as symptoms occur, in asymptomatic patients it is only recommended in cases of severe stenosis^1,2^. However, symptoms are prone to subjective interpretation and may not always be perceived by patients in the same way^29,30^. Additionally, monitoring moderate activity may also be beneficial in settings where physical activity is restricted to avoid symptoms. Koehler et al.^31^ have recently found that telemedical monitoring of heart failure patients can help to reduce all-cause mortality and hospital days due to unplanned cardiovascular reasons. The continuous monitoring of exercise capacity could additionally help to detect gradual clinical worsening and should, therefore, be further evaluated to better understand the potential benefits of such broadly available information for disease detection and therapy planning.

In cardiopulmonary exercise testing, exercise is usually quantified by an externally set workload. A heart rate-based approach, however, relies on the interpretation of a surrogate where literature evidence is used to identify activity states.

Finally, we acknowledge that this study has certain limitations. To determine resting heart rate, activity sensors data of the device were used. The paroxysmal occurrence of tachycardic atrial fibrillation, especially common in the older population^32^, may potentially influence the heart rate-based analysis of activity. To reduce the influence of atrial fibrillation on measurements, heart rate profiles have been scanned for tachycardia and if present, were excluded. However, it cannot be certain that datasets with short periods of atrial fibrillation may still be included. Additionally, moderate activity itself can trigger relevant tachycardia. The combined simultaneous use of heart rate sensors, actometers and novel integrated electrocardiographic sensors may help to better detect such events and to distinguish between tachyarrhythmia and activity.

The influence of age, medication and physical fitness on the resting heart rate may be a limitation for categorizing activity based on heart rate data. Therefore, the use of beta blockers was included as a covariate within the models and was shown to be without relevant impact for the achievement of target 6-minute walk distances or absolute distances. Furthermore, the thresholds for activity levels are based on individual resting heart rates. Consequently, for patients with low resting heart rates, e.g., due to beta blockers or high fitness level, the thresholds for activity levels would be correspondingly lower.

Compared to large-scale physical activity data^33^, the average number of steps per day in our cohort was high and may in parts be attributed to an increased awareness that can influence daily activity. Additionally, the large-scale datasets were acquired from mobile phones, that are not always carried. In contrast, the wrist-worn devices had a high wearing time, which has a direct impact on the number of recorded steps.

When determining exercise capacity based on daily physical activity, it should be considered that externals factors can affect activity. For example, exceptional situations or events occur during measurement periods, and thus daily activity can be influenced, not reflecting the individual exercise capacity. By extending measurement periods and patient counts, this confounder could be further limited. Several other factors including orthopaedic or mental diseases can influence everyday physical activity^34,35^. However, long-term changes in daily physical activity are commonly known to also result in changes in exercise capacity^36^. Higher sample sizes may help to further improve the model and reduce variances.

The 6-minute walk test has become a widely used prognostic marker of patient-centred outcomes including death or hospitalization^37^. Its ability to determine exercise capacity can be influenced by several individual physical and psychological factors, including pain, motivation and co-morbidities. Although it is considered to be a standardized test, its variability can be substantial even in healthy populations. As a goodness-of-fit indicator, *R*^2^ values of the robust regression model were approximately 0.5 within our cohort. In line with these findings, an inherently great amount of unexplainable variation can typically be found in exercise testing. In healthy subjects, coefficients of determination (*R*^2^) similar to our values have been reported when comparing the expected (equation-based reference values) and the actually achieved walking distances^38^. Additionally, the uncertainty of the model-based predictions (provided in Tables 3 and 4) overlapped with the minimal clinically important differences of the 6-minute walk test. Hence, we consider the findings in our disease-specific cohort to be clinically meaningful and vastly within the limitations of the 6-minute walk test^14^, although the amount of unexplained variation in this clinical standard method can be high.

Heart rate-based activity levels and the model’s output were not directly tested against more objective variables of functional capacity tests or morbidity/mortality outcomes. Therefore, further studies are needed to assess, how these measures translate into patient-specific outcomes. Some studies assessing 6-minute walk tests have found an association of results from cardiopulmonary exercise testing and the 6-minute walk test^39–41^, whereas others did not find the 6-minute walk test to be a reliable measure of exercise capacity^42^. Further studies testing the assessment of exercise capacity, using heart rate data obtained from wrist-worn devices, against cardiopulmonary exercise testing might be needed to support its validity. Furthermore, studies including other patient groups are needed to verify the method's applicability for other diseases. More datasets with a longer recording time could further reduce statistical uncertainty.

In summary, we were able to demonstrate that wrist-worn devices can be of use for predicting a patient's 6-minute walk test outcome, with uncertainties overlapping to the minimal clinically important differences and the MDC of the 6-minute walk test itself. The daily time spent in moderate activity was determined based on heart rate data obtained from these wearable devices. It was predictive for 6-minute walk distances and achievement of patient- (gender, age and BMI) specific target 6-minute walk distances, an established diagnostic and prognostic marker. Further studies in larger cohorts and a variety of disease groups are required to improve the method’s accuracy and to investigate if continuous recordings can provide helpful additional information for diagnostic processes and therapy planning.

## Methods

### Study population

The present study was part of the “EurValve” research initiative, focusing on decision support in patients with valvular heart disease. The project’s aim was to implement and test, in a relevant clinical target cohort, a decision support system (DSS) for aortic and mitral valve replacement and repair. The main component of the DSS was a combined 0D model of the cardiovascular system that includes modification options for valve repair and replacement, aiming to predict the haemodynamic effects of different types of treatment. The multi-centre study was conducted at three European sites in the Netherlands, the United Kingdom and Germany. In all recruited patients, clinical routine data were assessed and included demographics, anthropometrics, medical history and functional cardiac imaging. Patients with aortic stenosis, mitral regurgitation and mixed valvular heart disease recruited at Deutsches Herzzentrum Berlin (DHZB, German Heart Centre Berlin) and the Sheffield Teaching Hospitals were assessed using a Philips health watch (DL8791, Philips, Stamford, CT, USA). The device is licensed as a medical product in Europe. Heart rate as well as steps and activity counts from wrist movement was recorded.

The study protocol included wearing the device during everyday life for at least 23 h and performing a 6-minute walk test. Datasets were excluded if the heart rate-based analysis of daily activity was affected by tachycardic atrial fibrillation or if no 6-minute walk test was conducted. Patients were included at different stages and time points during the treatment process (before and/or after treatment).

The primary comparison was between the amount of time spent at different levels of activity each day, based on heart rate and 6-minute walk distances. Secondarily, daily activity was compared to the achievement of target 6-minute walk distances. All procedures followed the ethical guidelines of the 1975 Declaration of Helsinki and were approved by the local Ethical Committee at both sites (Ethikkomission Charité—Universtätsmedizin Berlin: EA2/093/16, NHS Health Research Authority: 17/LO/0283). Written informed consent was obtained from all included patients. Due to ethical regulations, untreated patients with severe aortic stenosis were not included at German Heart Centre Berlin. The study complied with the Strengthening the Reporting of Observational Studies in Epidemiology (STROBE) Statement. The study has been registered on ClinicalTrials.gov (NCT04068740).

### Six-minute walk test

The six-minute walk test was conducted before providing the patient with a device and was performed according to current guidelines^16^ shortly before the device recordings. The patient-specific target distance depending on gender, age and BMI was calculated using the established formula “6-minute walk distance = 1140 m − (5.61 × BMI) − (6.94 × age)” for men and “6-minute walk distance = 1017 m − (6.24 × BMI) − (5.83 × age)” for women^43^.

### Heart rate-based measurements of physical activity

The average heart rate as well as the total number of steps and activity counts were computed for each minute. Activity is a measure of accelerometery counts data obtained from the wrist-worn device. All data were analysed by an automated software tool to identify time spent at different levels of activity. Sleep and rest were determined based on steps and activity counts during recordings. Rest was defined as one or no steps per minute and an activity count below 475 arbitrary units (AU) per minute; sleep as an activity count below 70 AU.

The average heart rate during time spent resting while wearing the device was defined as the resting heart rate. All activity levels were then determined by changes in heart rate. Based on a meta-analysis of physiologic heart rate changes during different levels of activity^44^ and the identified resting heart rate, the level of activity was determined for each data point. An increase of the heart rate between 31 and 49 b.p.m. above resting heart rate was defined as light, between 50 and 88 b.p.m. as moderate and above 88 b.p.m. as high activity^44^.

### Statistical analysis

Continuous data are expressed as median and interquartile range (IQR, Q1–Q3) unless stated otherwise. Categorical data are presented as frequencies and percentages (%). Data distribution was tested using Shapiro–Wilk and Shapiro–Francia tests. A logistic regression model with continuous and binary patient-specific covariates (age, presence of aortic valve disease and presence of mitral valve disease, use of beta blockers) was used to evaluate the predictive quality of the percent time spent in moderate activity on binary outcome measures (achievement of target 6-minute walk distances). Additional to patient-specific anthropometric and demographic characteristics, both disease conditions were included as binary covariates to control for possible unobserved variables within the disease groups. The rationale for including these conditions in a single model instead of testing in separate models was the concept of general applicability of the method across different groups of valvular heart disease. Robust regression was used to assess multifactorial effects on 6-minute walk distances with consideration of outliers. Based on the robust regression, *R*^2^ values (as a goodness-of-fit measure) and predictive margins with standard deviations were calculated and plotted to visualize the combined effects of age, gender and BMI. Stata version 15.1 was used for statistical analysis. Interpretation of our findings followed the advice by the American Statistical Association^45^. MFPIGEN package was used to investigate the interaction between each pair of covariates and both predictive models were built without interactions.

### Reporting summary

Further information on research design is available in the Nature Research Reporting Summary linked to this article.

## Supplementary information

Reporting Summary

## Data Availability

The datasets analysed during the current study are available from the corresponding author on reasonable request.

## Code availability

The code used to process and analyse the findings of this publication may be made available to an investigator upon request for academic, research and non-commercial use.
